# The endoscopist’s third hand: external floss traction-assisted duodenal papillary intubation for hidden papilla

**DOI:** 10.1055/a-2612-3464

**Published:** 2025-06-18

**Authors:** Yan Zhang, Shanbin Wu, Yuping Zhang, Qing Yan, Guoliang Zhao

**Affiliations:** 1 Department of Gastroenterology, The First Affiliated Hospital of Shandong First Medical University and Shandong Provincial Qianfoshan Hospital, Jinan, Shandong, China

Difficult biliary or pancreatic cannulation during endoscopic retrograde cholangiopancreatography (ERCP) is among the leading causes of failed operations 1 . The adequate exposure of the ampullary orifice and reliable fixation of the duodenal papilla are always the basic principles of successful cannulation during ERCP 2 . Inspired by the application of traction in endoscopic treatment of early gastrointestinal tumors, we have developed external floss traction to accomplish difficult cannulation during ERCP.

A 70-year-old man was referred for ERCP for suspected common bile duct stones. After insertion of a therapeutic duodenoscope, redundant duodenal folds became visible in the descending duodenum, initially giving rise to a “hidden papilla” situation. We developed the external floss traction-assisted duodenal papillary intubation method to effectively accomplish successful biliary cannulation and stone extraction ( Video 1 ).

External floss traction-assisted duodenal papillary intubation for hidden papilla.Video 1

Attempts were made to access the biliary tree using the guidewire-aided cannulation technique; however, as the ampulla of Vater was hidden under duodenal folds, attempts to gain access were unsuccessful ( Fig. 1 ). Subsequently, the external floss traction method was applied to pull the duodenal folds ( Fig. 2 ), and the ampullary orifice became visible ( Fig. 3 ). Successful cannulation of the bile duct was finally achieved ( Fig. 4 ), and bile duct sand-like stones were successfully removed ( Fig. 5 ). Then, a nasobiliary drainage tube was placed smoothly into the bile duct, with fluent drainage of bile juice.

**Fig. 1:**
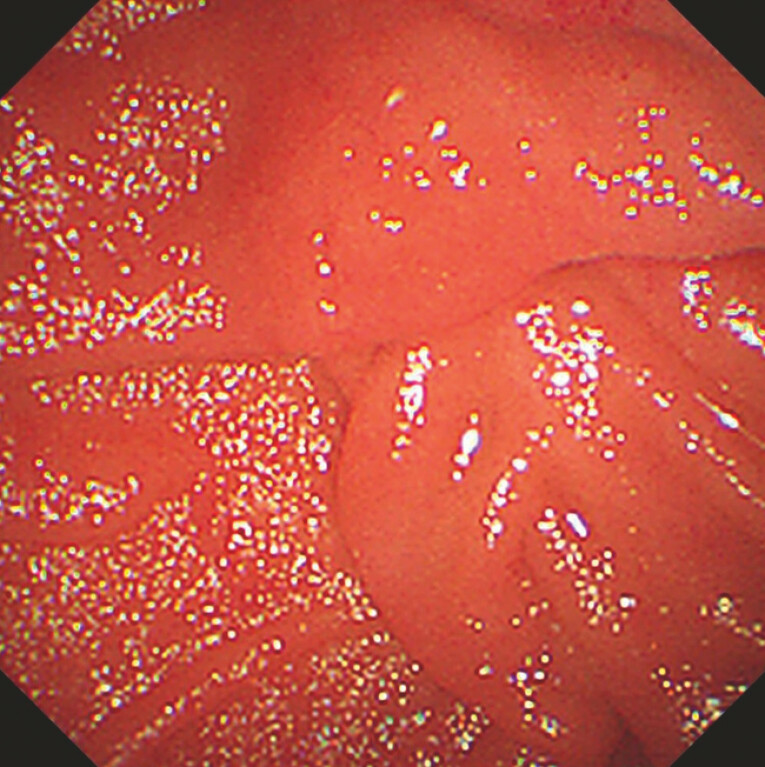
Redundant duodenal folds became visible in the descending duodenum, initially giving rise to a “hidden papilla” situation.

**Fig. 2:**
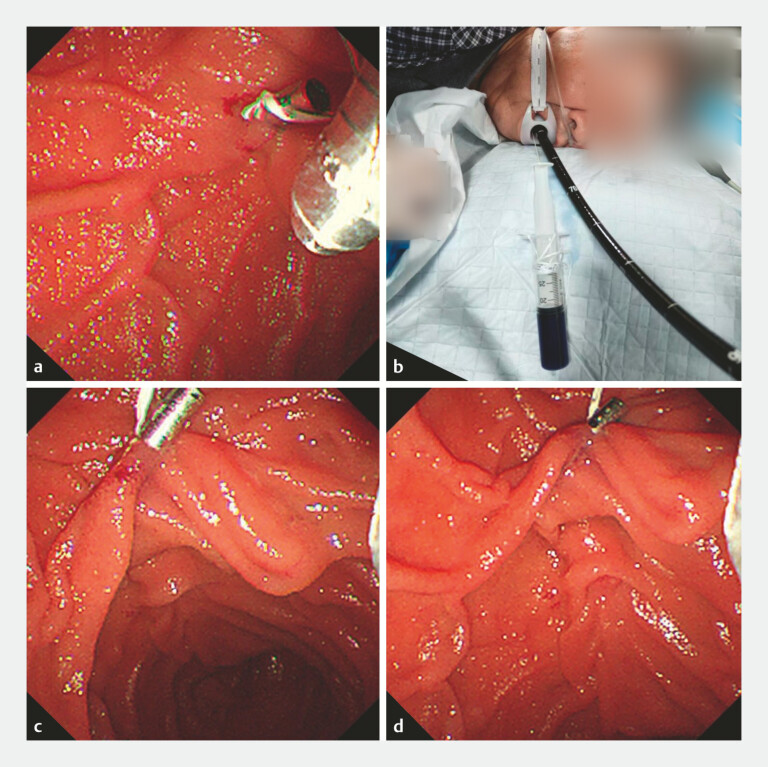
The external floss traction method. **a** The floss was fixed to the clip, and the clip was applied to the duodenal fold. **b** The floss was secured externally and was pulled to apply traction. **c, d** After external floss traction, the ampullary orifice became visible.

**Fig. 3:**
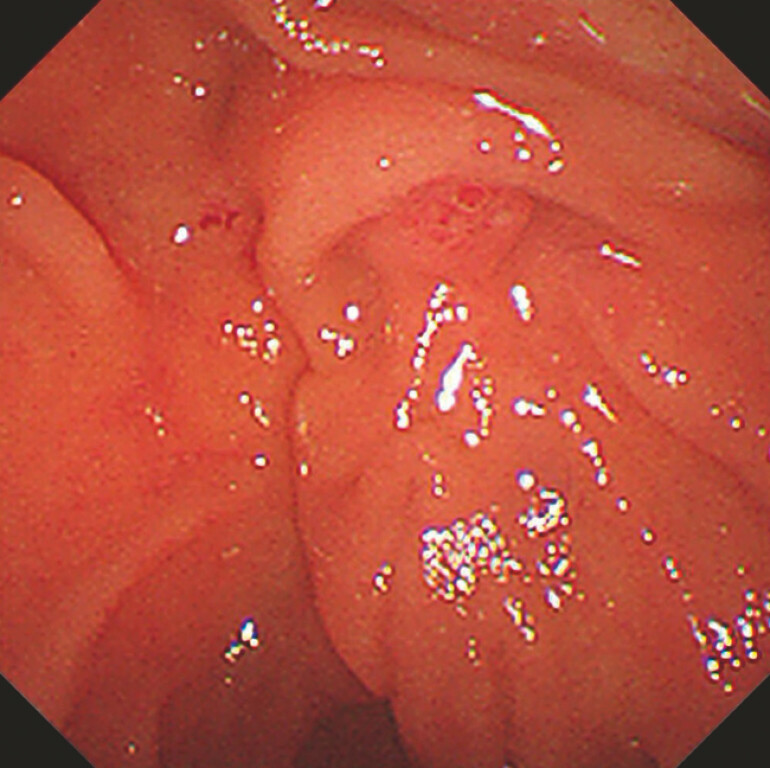
The visible ampullary orifice.

**Fig. 4:**
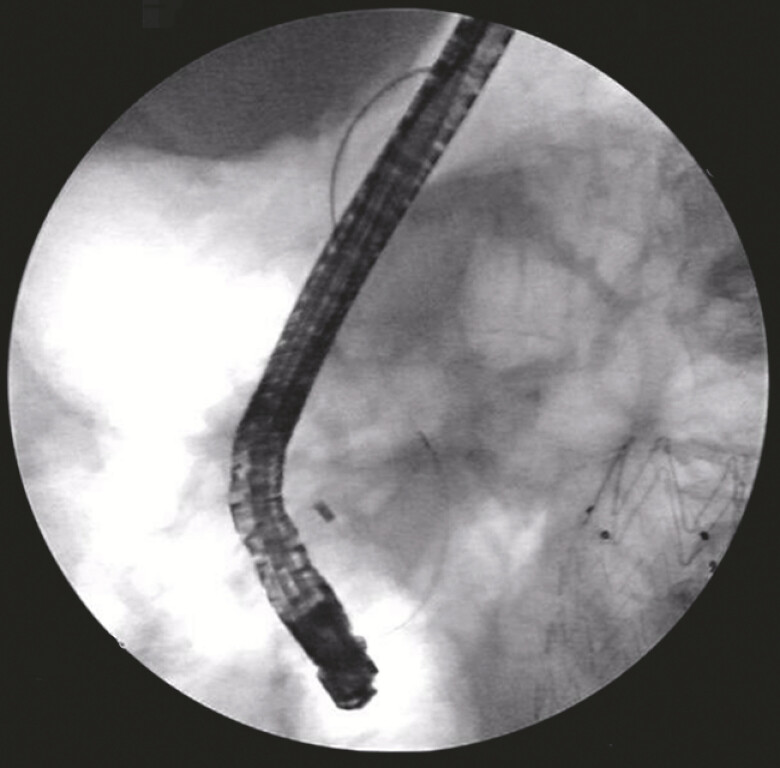
Successful cannulation of the bile duct was finally achieved.

**Fig. 5:**
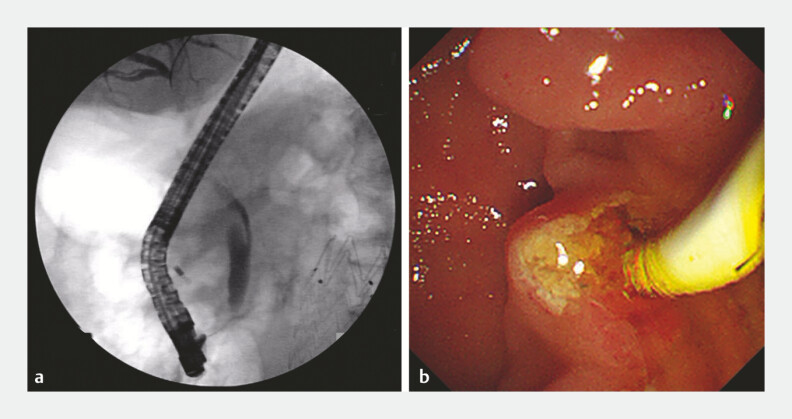
Successful removal of bile duct stones. **a** Cholangiography showed no significant filling defect. **b** A small number of sand-like stones were removed by the lithotomy balloon.

In this patient, the technique of external floss traction was employed to optimize the view of the papilla, and led to successful biliary cannulation in a short time. As the external floss traction method does not interfere with ERCP instruments and is inexpensive, easily accessible, and simple and convenient to manipulate, it may be applied to routine biliary cannulation in any patient where a traction technique is required.

Endoscopy_UCTN_Code_TTT_1AR

## Notes

Endoscopy E-Videos https://eref.thieme.de/e-videos
*E-Videos* is an open access online section of the journal *Endoscopy* , reporting on interesting cases and new techniques in gastroenterological endoscopy. All papers include a high-quality video and are published with a Creative Commons CC-BY license. Endoscopy E-Videos qualify for HINARI discounts and waivers and eligibility is automatically checked during the submission process. We grant 100% waivers to articles whose corresponding authors are based in Group A countries and 50% waivers to those who are based in Group B countries as classified by Research4Life (see: https://www.research4life.org/access/eligibility/ ). This section has its own submission website at https://mc.manuscriptcentral.com/e-videos .
